# Fatty acids composition of aorta and saphenous vein tissues in patients with coronary artery diseases

**DOI:** 10.15171/jcvtr.2017.13

**Published:** 2017-06-12

**Authors:** Mirhamid Paytakhti Oskouei, Masoud Pezeshkian, Naser Safaie, Masoud Darabi, Amir Fattahi, Hossain Bijanpour, Maghsod Shaaker, Ahmad Reza Jodati, Mohammad Nouri

**Affiliations:** ^1^Cardiovascular Research Center, Tabriz University of Medical Sciences, Tabriz, Iran; ^2^Department of Clinical Biochemistry, School of Medicine, Tabriz University of Medical Sciences, Tabriz, Iran

**Keywords:** Coronary Artery Disease, Fatty Acid, Atherosclerosis, Aorta, Saphenous Vein

## Abstract

***Introduction:*** Considering importance of fatty acids in developing coronary artery disease (CAD) and lack of information about saphenous vein which is commonly used as coronary arterial bypass, in this study we investigated differences in fatty acids composition between saphenous vein and aorta tissues in patients with CAD.

***Methods:*** Biopsy samples of aortic tissues and saphenous veins as well as blood samples were obtained form 42 patients with CAD. Fatty acids composition of the tissues was determined using gas chromatography and also serum lipid profile was evaluated by commercial kits.

***Results:*** Levels of palmitic acid (16:0) were significantly higher in aorta in compared with saphenous (*P *< 0.001). Also levels of most unsaturated fatty acids (16:1, 18:1n-9, 18:1t, 18:2t, 18:3 n‐9 and 22:3n-3) were statistically higher in saphenous tissue than aorta tissue (*P *< 0.05). Mean levels of linoleic acid (18:2 n-6) was higher in aorta tissue in comparison with saphenous tissue (*P *= 0.01). We observed positive correlations between serum levels of LDL-C with elaidic acid and linoleic acid levels in saphenous. Evaluation of aorta tissue fatty acids revealed that palmitoleic acid (16:1) had positive and arachidonic and linoleic acids had negative correlations with serum HDL-C levels.

***Conclusion:*** Our results revealed difference between fatty acids composition of aorta and saphenous vein tissues and existence of correlations between the fatty acids levels with serum lipid profile. The saphenous vein had higher poly-unsaturated fatty acids in compared to aorta tissue and thus this vein is not at risk of atherosclerosis and can be used as coronary arterial bypass.

## Introduction

One of the main causes of mortality in the world is cardiovascular diseases (CVD) that the endothelial dysfunction is primary event in CVD developing.^1^ Endothelial dysfunction is associated with impairment in anti-inflammatory and anti-coagulant properties of the endothelium as well as vascular growth and consequently vasoregulatory malfunctions.^2,3^ Vascular endothelium has a crucial role in the regulation of platelet activity, vascular tone and blood flow.^2^ Recent studies suggest that endothelial dysfunction is an early indicator for atherosclerosis and can be noticed before appearing of vessel wall structural alterations in angiography or ultrasound.^4^ It has been suggested that atherosclerosis is preventable by decreasing lipid accumulation in the arterial wall through improving lifestyle such as avoidance of tobacco smoking, healthy eating and doing exercise.^5,6^ One of the main surgical treatments in the selected patients with coronary artery disease (CAD) is coronary artery bypass grafting (CABG).^7^ Although surgical techniques has been well improved but still there is lots of problem in choosing appropriate vein or artery grafts and inhibiting the grafts failure as it was documented that in first year about 15% and in 10 years about half of venous graft occlude or fail.^7-9^ Although there is some studies on selecting appropriate vein as well as epidemiological and pathological causes of venous graft failure, but these issues still remains challenging.^7^

Many studies have been conducted to investigate lipid accumulation in the arterial wall of patients with atherosclerosis but many questions about mechanisms of dyslipidemia and progression of atherosclerotic lesions have been remained unanswered.^10^ One of the unclear issues in atherosclerosis is exact role of various local fatty acids. Although many studies have evaluated effects of dietary fatty acids as well as plasma levels of them on coronary atherosclerosis,^11,12^ but there is no enough information about fatty acids content and their possible roles in the tissues which are involved in the atherosclerosis.

It has been reported that cholesterol as one of the major lipids in atherosclerotic plaques which possesses fatty acids, enters from the blood rather than in situ synthesis.^13,14^ However existence of higher free cholesterols than cholesterol esters in the plaques^15^ which is in contrary with the finding about high ratio of cholesterol esters/free cholesterol in plasma,^16^ led to arise theory of coming the lipids from vessels growing into the plaque.^17^ On the other hand higher levels of fatty acid synthesis in atherosclerotic arteries in compared with intact arteries highlights possible role of fatty acid metabolism in tissues which are involved in atherosclerosis.^18^

In a study it has been reported that the fatty acids composition of coronary arteries was significantly different between sudden cardiac death and traffic accident victims.^19^ Also Bahrami et al^20^ have demonstrated lower levels of unsaturated fatty acids and higher amounts of saturated fatty acids in atherosclerotic aorta in compared with the internal mammary arteries. Furthermore different fatty acids composition of aorta artery and adipose tissue between patients with and without CAD has been shown previously.^21^ These finding emphasize on importance of local fatty acids composition in developing atherosclerosis and CAD. However there is no information about fatty acids composition of saphenous vein which is commonly used as coronary arterial bypass and also has structural differences with arteries.^22^ The aim of this study was to investigate differences in fatty acids composition between saphenous vein and aorta tissues, as well as associations between the fatty acids levels with serum lipid profile in patients undergoing CABG.

## Materials and Methods

### Participants

Frothy-two individuals (mean age: 53.2±7.5 years), documented as CAD patients were included in this study. All patients had at least two vessels coronary artery disease (≥50% stenosis) based on the angiographic data and were scheduled for CABG at Shahid-Madani heart hospital in Tabriz, a northwest city of Iran. Also we recruited patients with body mass index (BMI) ≤34.9 kg/m^2^ and the patients with class II and III obesity (BMI ≥39 kg/m^2^) were not involved. The exclusion criteria were: having acute inflammation, auto-immune diseases, kidney and liver diseases, diabetes, anemia or malignant tumors. Also patients with prior CABG as well as aortic or mitral valve replacement were excluded from the study.

### Sample collection and preservation

Biopsy samples (1-2 g) were obtained during the surgery from aortic tissues and saphenous veins and then were dissolved in hexane and kept frozen at - 70°C in glass vials for fatty acid measurement. Also in order to lipid profile analysis, approximately 5 mL of venous blood samples in the fasting state at 8:00 am were taken from all participants and the serums were separated using centrifugation.

### Fatty acids analysis

Fatty acids were extracted using the Bligh and Dyer method^23^ and the percentage of each fatty acid was determined using gas chromatography. In brief, the solution of fat extract in hexane was evaporated under a stream of nitrogen to near dryness, and the lipids were esterified with methanol during catalysis with acetyl chloride. Then fatty acid methyl ester derivatives were separated on a 60- x 0.25-mm Teknokroma TR-CN100 column using gas chromatograph system (Buck Scientific model 610, USA) equipped with a split injector and a flame ionization detector. The oven temperature program was 170-210°C, 1°C/min, and then isothermal for 45 minutes. The combined inter- and intra assay of variation was 2.1% for palmitic acid (16:0), 2.7 for linoleic acid (18:2n-6), 18.6 for eicosapentaenoic acid (EPA), and 11.2 for docosahexaennoic acid (DHA). The known fatty acids from Sigma chemicals was employed as standards to peak retention times identification. The levels of fatty acids were calculated as the percentage of each fatty acid of the total.

### Serum lipid profile analysis

The levels of triglycerides (TG), total cholesterol (TC) and high density lipoprotein-cholesterol (HDL-C) determined by enzymatic colorimetric method (Pars Azmoon Co, Tehran, Iran) with an automated chemical analyzer (Abbott analyzer, North Chicago, IL, USA). Low-density lipoprotein-cholesterol was calculated using the Friedewald formula.

### Statistical analyses

The data are reported as mean ± standard deviation (SD). Normal distribution of the data was confirmed by Kolmogorov-Smirnov test. The paired *t* test was used to compare fatty acids levels between saphenous vein and aorta tissues in each patient. Also the Pearson correlation test was applied to investigate possible associations between the fatty acids levels with serum lipid parameters. *P* values <0.05 were considered significant. SPSS V.16 software was used for the statistical analysis.

## Results

Demographic and clinical characteristics of the studied population are shown in Table 1. Most of the recruited patients were male (about 80%) and only 4% of them were smoker. Among studied population, 9% of them had family history of CVD.

**Table 1:** Demographic and clinical characteristics of the studied population

**CVD patients (n = 42)**
Age (y)	53.2±7.5
Body mass index (kg/m^2^)	27.5±4.4
Gender (n)	
Male	34
Female	8
Smoking (%)	4
High blood pressure (%)	30
Family history of CVD (%)	9
Consumption of hydrogenated vegetable oil (%)	60
Medications (%)	
Aspirin	38
Statins	25
β-Blockers	35
Calcium antagonists	7

Abbreviation: CVD, cardiovascular disease.

In studied patients mean levels of total cholesterol was higher than normal range (214 ± 45 mg/dL) and mean levels of triacylglycerol, 178 ± 50 mg/dL, was borderline high (Table 2). Also the patients had low levels of HDL-C but the mean level of LDL-C was almost close to ideal (Table 2).

**Table 2:** Lipid profile of patients with cardiovascular disease

**CVD patients (n = 42)**
Total cholesterol (mg/dL)	214±45
Triacylglycerol (mg/dL)	178±50
HDL-C (mg/dL)	35±9
LDL-C (mg/dL)	112±24
Triacylglycerol/HDL	3.6±2.1

Abbreviations: HDL-C, high density lipoprotein-cholesterol; LDL-C, low density lipoprotein-cholesterol; CVD, cardiovascular disease.

Using gas-liquid chromatography, we analyzed saphenous and aorta tissues fatty acids composition. As shown in table 3, levels of saturated fatty acids were not different between these tissues and just levels of palmitic acid (16:0) were significantly higher in aorta in compared with saphenous tissue (*P *< 0.001). Levels of most unsaturated fatty acids (16:1, 18:1n-9, 18:1t, 18:2t, 18:3 n‐9 and 22:3n-3) were statistically higher in saphenous tissue than aorta tissue (*P *< 0.05). On the other hand, mean levels of linoleic acid (18:2 n‐6) was higher in aorta tissue in comparison with saphenous tissue (*P *= 0.01; Table 3).

**Table 3:** Fatty acids composition of saphenous and aorta tissues in patients with cardiovascular disease

**% Of total fatty acids**	**Aorta**	**Saphenous**	***P *** **value**
12:0 (Lauric acid)	1.02±0.52	0.9±0.46	0.265
14:0 (Myristic acid)	3.1±1.1	3.5±1.5	0.167
16:0 (Palmitic acid)	28.9±3.9	20.4±3.2	<0.001
16:1t (Palmitoleic acid)	1.3±0.4	1.4±0.34	0.220
16:1 (Palmitoleic acid)	5.1±3.1	7.2±3.3	0.0035
18:0 (Stearic acid)	5.9±2.8	6.7±2.1	0.142
18:1n-9 (Oleic acid)	22.2±6.3	28.1±5.8	<0.001
18:1 t (Elaidic acid)	5.7±1.9	8.3±2.8	<0.001
18:2 t (Linoleic acid)	1.6±0.8	2±0.8	0.0245
18:2 n-6 (Linoleic acid)	13.1±2.67	11.1±4.2	0.01
18:3 n-9 (Linolenic acid)	4±1.6	5.5±1.9	<0.001
20:4n-6 (Arachidonic acid)	0.6±0.32	0.58±0.2	0.732
20:5 n-3 (Eicosapentaenoic acid)	0.14±0.08	0.14±0.06	1
22:3 n-3 (Docosatrienoate acid)	1.5±0.8	2.1±1.4	0.018
22:6 n-3 (Docosahexaenoic acid)	0.17±0.09	0.17±0.08	1

The results of correlation analysis of fatty acids levels in saphenous or aorta tissues with lipid profile of serum were listed in Table 4. We found that serum HDL-C levels were positively and negatively correlated with levels of myristic acid (14:0) and linoleic acid (18:2 n‐6) in saphenous tissue, respectively. Moreover we observed positive correlations of elaidic acid (18:1 t) and linoleic acid (18:2 n‐6) levels in saphenous with serum levels of LDL-C. Elaidic acid (18:1 t) of saphenous also had positive correlation with total cholesterol levels. Evaluation of aorta tissue fatty acids revealed that palmitoleic acid (16:1) had positive and arachidonic (20:4n-6) and linoleic (18:2 n‐6) acids had negative correlations with serum HDL-C levels. Also in this tissue myristic acid (14:0) was negatively correlated with TG/HDL-C ratio.

**Table 4:** Correlations between various fatty acids levels in saphenous and aorta tissues with serum lipid profile in patients with cardiovascular disease

	**TC**	**TG**	**HDL-C**	**LDL-C**	**TG/HDL-C**
	r	r	r	r	r
**Saphenous tissue**					
12:0 (Lauric acid)	0.05	0.2	0.18	0.05	0.08
14:0 (Myristic acid)	-0.05	0.01	0.45*	0.16	-0.23
16:0 (Palmitic acid)	0.01	0.10	0.22	-0.15	0.01
16:1t (Palmitoleic acid)	-0.1	0.05	0.14	-0.21	0.04
16:1 (Palmitoleic acid)	-0.02	-0.11	0.3	-0.19	-0.35
18:0 (Stearic acid)	-0.05	-0.05	-0.3	0.01	0.24
18:1n-9 (Oleic acid)	-0.25	0.12	-0.01	0.01	0.2
18:1 t (Elaidic acid)	0.35*	0.08	-0.3	0.5**	0.3
18:2 t (Linoleic acid)	0.15	-0.09	-0.1	0.19	-0.1
18:2 n-6 (Linoleic acid)	0.3	-0.14	-0.51**	0.55**	0.25
18:3 n-9 (Linolenic acid)	0.2	-0.29	-0.14	0.12	0.08
20:4n-6 (Arachidonic acid)	-0.2	-0.1	-0.25	-0.09	0.1
20:5 n-3 (Eicosapentaenoic acid)	-0.19	0.1	0.2	-0.15	0.15
22:3 n-3 (Docosatrienoate acid)	0.12	0.09	0.14	0.19	-0.15
22:6 n-3 (docosahexaenoic acid)	-0.25	0.08	-0.24	-0.27	0.14
**Aorta tissue**					
12:0 (Lauric acid)	0.04	-0.24	-0.02	-0.1	-0.17
14:0 (Myristic acid)	0.04	-0.2	0.21	0.07	-0.45*
16:0 (Palmitic acid)	-0.02	0.1	0.26	-0.14	-0.2
16:1t (Palmitoleic acid)	0.12	-0.23	0.09	0.18	-0.23
16:1 (Palmitoleic acid)	0.14	0.14	0.45*	-0.05	-0.19
18:0 (Stearic acid)	0.09	-0.04	-0.3	0.18	0.23
18:1n-9 (Oleic acid)	-0.08	0.14	-0.23	-0.06	0.28
18:1 t (Elaidic acid)	-0.08	-0.1	0.19	-0.9	-0.21
18:2 t (Linoleic acid)	0.2	0.16	-0.19	0.31	0.21
18:2 n-6 (Linoleic acid)	0.09	-0.08	-0.54**	0.24	0.23
18:3 n-9 (Linolenic acid)	0.14	-0.01	-0.02	0.16	-0.1
20:4n-6 (Arachidonic acid)	0.08	0.1	-0.46*	0.11	0.18
20:5 n-3 (Eicosapentaenoic acid)	0.1	0.26	-0.29	0.09	0.26
22:3 n-3 (Docosatrienoate acid)	0.1	0.25	-0.28	0.1	0.27
22:6 n-3 (docosahexaenoic acid)	-0.1	0.18	0.14	-0.2	0.15

Abbreviations: HDL-C, high density lipoprotein-cholesterol; LDL-C, low density lipoprotein-cholesterol; TC, total cholesterol; TG, triacylglycerol. *P < 0.05 and ** *P* < 0.001.

## Discussion

CADs are accompanied by endothelial dysfunction^4^ and beneficial effects of dietary unsaturated fatty acids have been reported previously.^24,25^ Saturated and mono- or poly-unsaturated fatty acids, as dietary intake compounds, have wide variety of physiological processes through their role in the prevention, treatment and pathogenesis of CVD.^11,26^ Epidemiological and clinical studies are now attended on elucidating the exact action mechanisms of these compounds on vascular system, especially in prevention of atherosclerosis. Considering saphenous vein as the most common coronary arterial bypass and also reports about this vessel structural differences with arteries^22^ in this study we evaluated fatty acids composition of saphenous vein and aorta tissues in patients undergoing CABG.

As it was expected, the studied patients had high serum levels of LDL-C, TC, TG and triacylglycerol/HDL ratio and also the HDL-C levels were low. Such lipid profile is so common in CAD patients^27^ and is considered as CAD risk factor.^28,29^

The assessment of fatty acids contents in saphenous vein and aorta of CAD patients indicated that the fatty acids composition between these vessels was different. We found higher concentration of palmitic acid as saturated fatty acid in aorta in compared with saphenous vein (*P*<0.001). Comparing the mono-unsaturated fatty acids levels between saphenous and aorta demonstrated that levels of palmitoleic acid and oleic acid as well as its *trans* isomer (elaidic acid) were significantly lower in aorta than saphenous vein. The poly-unsaturated fatty acids levels were also different between the two vessels as we observed significantly lower proportions of the *trans* isomer of linoleic acid (18:2 t), linolenic acid (18:3 n-9) and docosatrienoate acid (22:3 n-3) in aorta in compared to the saphenous. Based on our knowledge, there is no report on differences of fatty acids composition between saphenous and aortic tissues of CAD patients. However, in accordance with our results, in previous studies higher levels of palmitic acid and also lower levels of poly-unsaturated fatty acids in phospholipids of aorta and coronary arteries of patients with CAD in compared with control individuals have been reported.^19,21^ Moreover in a study on fatty acids composition of aorta and internal mammary of patients with CAD same as our results, lower levels of unsaturated fatty acids (oleic acid, elaidic acid, linolenic acid and docosatrienoic acid) and higher percentage of palmitic acid in aorta samples than internal mammary have been reported.^20^ Moreover they also found statistically different levels of stearic acid (18:0), arachidonic acid (20:4n-6) and eicosapentaenoic acid (20:5-n3) between aorta and internal mammary arteries.^20^ We did not find such differences between aorta and saphenous vein maybe due to structural differences between saphenous vein and internal mammary artery.

Interestingly, in the present study increased levels of linoleic acid (18:2 n-6) were found in aorta tissue as compared to the saphenous vein. Higher levels of linoleic acid in aorta and coronary arteries of individuals with CAD compare to non-CAD subjects and also in aorta in compared with internal mammary artery in patients with CAD have been shown previously.^19-21^ Considering the previous reports about existence of positive association between the grade of atherosclerosis and linoleic acid percentages in phospholipids of coronary arteries,^19^ it could be postulated that such an increase in our study could be due to aortic atherosclerosis. In support of this hypothesis positive correlation between linoleic acid intake and new atherosclerotic lesions formation has been demonstrated.^30^ Another explanation for higher levels of linoleic acid and lower levels of other unsaturated fatty acids in aortic tissue comparing saphenous vein can be reduced activity of delta-6-desaturase in aorta as has been suggested previously.^19^ The ω6 fatty acids such as linoleic acid as proinflammatory factor can activate vascular endothelial cells and consequently may cause atherosclerosis.^31,32^

Such differences between fatty acids composition of aorta artery and saphenous vein could be partly due to structural differences between arteries and veins but some part of these differences could also be because of atherosclerosis and cardiovascular complications that the aorta was involved. Reports about different fatty acids composition between aorta and internal mammary of patient with CAD confirm this possibility.^20^ However there is no enough information about pattern of fatty acids modification and its role in atherosclerosis pathogenesis.

In this study we detected inverse correlations between serum HDL-C with linoleic acid levels in saphenous and aorta tissues and also with arachidonic acid levels of aortic tissue. Besides, linoleic acid levels in saphenous vein were positively correlated with LDL-C in serum. The negative correlation between HDL-C and arachidonic acid could be explained by results of previous study reported that in CAD patients, HDL-hypocholesterolemia decreases ability of platelet to produce nitric oxide (NO) and consequently reduction in NO levels itself inhibits arachidonic acid metabolism. NO induces the inhibitory influence through various direct and indirect ways such as inhibition of cyclooxygenase activities^33^ and thromboxane A2 production.^34^ In our previous study we also found negative correlations between levels of linoleic acid in epicardial and subcutaneous adipose tissues with serum levels of HDL-C and a positive correlation between this fatty acid content in subcutaneous adipose tissue with serum LDL-C in patients with CAD.^35^ There are wide evidences that the HDL-C and LDL-C are protective and risk factors for atherosclerosis and its complications, respectively^36,37^ and on the other hand linoleic and arachidonic acids have been documented as atherogenic fatty acids which cause inflammation and platelet aggregation.^31,32^ So finding the associations between these fatty acids with LDL-C and HDL-C seems reasonable. Moreover, we observed a positive correlation between the HDL value and palmitoleic acid in aorta which confirms the protective effect of palmitoleic acid as MUFAs against CAD.^38,39^

It has been demonstrated that elaidic acid (18:1t) is cholesterol elevating factor.^40^ Moreover results of a 25-year follow up study conducted in seven countries, have been showed positive correlation between dietary intake of elaidic acid and serum cholesterol.^41^ These reports confirm our finding about existence of positive correlation between elaidic acid in saphenous and serum total cholesterol.

Considering the association between fatty acids composition of aorta and saphenous tissues with serum lipid profile that was found in the present study, it can be concluded that composition of dietary fatty acids which has been suggested as important factor for CAD development^42,43^ exerts its effects partly via changing serum lipid profile and consequently vessel wall fatty acids composition. Although observed differences in fatty acids composition between aorta and saphenous vein tissues and also between aorta tissues of CAD and NON-CAD patients^19,21^ as well as aorta and internal mammary arteries of CAD patients^20^ emphasize that in addition to diet, local lipid metabolism and existence of atherosclerosis can be determinative factors for fatty acid composition of vessel walls. However the ways that different dietary intake or lipid metabolism could influence tissue fatty acids composition still remain to be elucidated. However, in our study we did not have information about dietary habits and life-style which could possibly affect the fatty acids composition of the tissues. Although it should be noted that in the present study the comparisons were done between two tissues of each patient, so any confounding factor such as dietary habits which could affect the evaluated parameters, affects the parameters of both saphenous and aorta tissues in same patient and consequently could not affect the overall result of our study. Another limitation of our study was lack of proper control without CVD because of impossibility of taking such samples from healthy individuals. However further studies could be conducted on immediately dead persons without CVD or animal models.

## Limitations

In this study due to the unavailability of healthy individuals saphenous and aorta tissues we could not compare the results with control. Also due to difficulty in finding patients our study was conducted with almost small sample size. Another limitation of our study was lack of information about diet of patients as it can has effect on fatty acids composition of saphenous and aorta tissues.

## Conclusion

In conclusion our results revealed difference between fatty acids composition of aorta and saphenous vein tissues in CAD patients and existence of correlations between the fatty acids levels and serum lipid profile. Owing our results about higher levels of PUFAs in saphenous vein compared to aorta tissue, it is likely that the vein has almost low risk for atherosclerosis and may be able to be used as coronary arterial bypass.

## Competing interests

All authors declare no competing financial interests exist.

## Ethical approval

Approval for this study was granted by the ethical committee of Tabriz University of Medical Sciences and the informed written consent was obtained from all participants (IRB cod: IR.TBZMED.REC.1395.1316; date of approval: 2016)

## Acknowledgments

The work was supported by a grant from the Cardiovascular Research Center, Tabriz University of Medical Sciences. This article was extracted from the M.Sc. thesis of Mirhamid Paytakhti Oskouei.
